# Diagnosis and treatment of pediatric food allergy: an update

**DOI:** 10.1186/s13052-014-0108-0

**Published:** 2015-02-19

**Authors:** Pasquale Comberiati, Francesca Cipriani, Alina Schwarz, Daniela Posa, Cristina Host, Diego G Peroni

**Affiliations:** Pediatric Clinic, Department of Life and Reproduction Sciences, University of Verona, Verona, Italy; Pediatric Unit, Department of Medical and Surgical Sciences, University of Bologna, Bologna, Italy; Department of Pediatric Pneumology and Immunology, Charité Medical School, Berlin, Germany; Dipartimento Riproduzione e Accrescimento, Sezione di Pediatria, Azienda Ospedaliero-Universitaria di Ferrara, Via A. Moro 8, Cona, 44124 Ferrara Itali; University of Ferrara, Section of Paediatrics, Corso Giovecca 203, 44100 Ferrara, Italy

**Keywords:** Anaphylaxis, Avoidance, Children, Component-resolved diagnosis, Food allergy, Specific oral tolerance induction

## Abstract

The prevalence of pediatric food allergy and anaphylaxis has increased in the last decades, especially in westernized countries where this emerging phenomenon was marked as a “second wave” of the allergic epidemic. Over recent years great advances have been achieved in the field of *in vitro* allergy testing and component-resolved diagnosis has increasingly entered clinical practice. Testing for allergen components can contribute to a more precise diagnosis by discriminating primary from cross-reactive sensitizations and assessing the risk of severe allergic reactions.

The basic concept of the management of food allergy in children is also changing. Avoidance of the offending food is still the mainstay for disease management, especially in primary health care settings, but it severely affects the patients’ quality of life without reducing the risk of accidental allergic reactions. There is a growing body of evidence to show that specific oral tolerance induction can represent a promising treatment option for food allergic patients. In parallel, education of food allergic patients and their caregivers as well as physicians about anaphylaxis and its treatment is becoming recognized a fundamental need. International guidelines have recently integrated these new evidences and their broad application all over Europe represents the new challenge for food allergy specialists.

## Introduction

The prevalence of pediatric food allergy (FA) and anaphylaxis has increased in the last decades, with westernized countries experiencing the highest rates. This emerging phenomenon marked as a “second wave” of the allergic epidemic has raised concern among the scientific community, which is currently investigating on potential risk factors [1-5]. Pediatric allergists are also experiencing remarkably changes in the pattern of allergic sensitization and disease manifestations, with a wider range of allergenic foods and increase in non-IgE-mediated gastrointestinal disorders [6].

The cornerstone in the diagnostic workup of FA is the oral food challenge (OFC) which is time and cost-consuming and involves the risk of adverse allergic reactions. To this purpose, there is growing interest concerning *in vitro* allergy diagnostic tests to reduce the need for OFC [7].

FA treatment has also emerged as a developing research field over the past decades. Indeed, the current management of FA comprises a strict avoidance of triggering foods, which has significant negative impact on nutritional, psychological and economic status and poses the risk of developing allergic reactions after accidental exposure [8].

This review examines the existing relevant literature focusing on new diagnostic and therapeutic strategies for FA in children.

### Search strategy

References were identified by searches of MEDLINE, PubMed and online Cochrane databases. The terms searched were food allergy and all of the following, separately and in combination: diagnosis/treatment/ management/avoidance/anaphylaxis/component-resolved diagnosis/molecular allergy/oral tolerance/oral immunotherapy/oral tolerance induction. We only searched for English-language original studies, reviews and commentaries conducted on children aged 0-18 years. Manuscripts published until November 2014 were included.

### Diagnostic procedures are improving through molecular approaches

According to the current guidelines the basic approach to diagnosis of FA firstly includes a detailed clinical history and physical examination, which should drive the choice of the most appropriate allergy diagnostic tests [8,9]. Skin prick test (SPT) and serum-specific Immunoglobulin E (sIgE) for food allergens are the first-line tests to assess an IgE-sensitization, but their low positive predictive value makes the elimination diet and OFC necessary to confirm the diagnosis [10]. OFC, particularly the double-blind placebo-controlled food challenge, is still considered the reference standard test for diagnosis of IgE-mediated and non-IgE-mediated FA [11]. Performing OFC can better define the real prevalence of FA and improve patients’ quality of life by preventing unnecessary elimination diet [8]. However, OFCs are costly and time-consuming, require a specialized setting and team, and involve the risk of developing severe allergic reactions, which can be stressful for both patients and families [12,13].

Several studies have proposed threshold-levels of SPT and sIgE to predict a positive outcome in OFC [14,15]. Such cut-off values are influenced by the characteristics of both population examined and methodologies used and may not be generalizable to other populations, as recently reviewed by Peters et al. for SPT to egg and peanut [16]. A recent Dutch study showed that a positive outcome in OFC can be predicted by using a multivariate model risk score, which considers the provocative food, the time between allergen ingestion and development of symptoms and sIgE level [17]. Over recent years great advances have been achieved in the search for safe and reliable *in vitro* tests to reduce the need for OFC. To this purpose, the measurement of sIgE to allergenic molecular components from allergenic sources, namely component-resolved diagnosis (CRD), has increasingly entered clinical practice [18]. CRD can contribute to identify triggering allergens by discriminating primary from cross-reactive sensitization in poly-sensitized patients. Moreover CRD can enable the risk assessment of severe allergic reactions leading to an improved patient management [18].

- **Peanut and tree nut allergy -** The major peanut allergen components Ara h 1, Ara h 2 and Ara h 3 are storage protein [19]. Ara h 8 is a member of the pathogenesis-related protein family (PR-10), which is relevant to patients with birch pollen allergy [20]. Ara h 9 is a nonspecific lipid transfer protein (nsLTP) and has recently been reported to be an important allergen in the Mediterranean area [21]. Ara h 2 has been highlighted as marker of primary sensitization, persistent allergy and severe reactions [22]. In a study of 181 French children with suspected peanut allergy, Ara h 6 and Ara h 2 were the best predictors of peanut allergy [23]. In a multicentre prospective study of 210 German children with suspected peanut or hazelnut allergy, sIgE to Ara h 2 and Cor a 14 resulted more reliable in predicting outcomes in OFC than sIgE to peanut or hazelnut extracts [24]. Similarly, in a recent Dutch study including 161 hazelnut-sensitized adult and children, sIgE to Cor a 9 and Cor a 14 were strongly associated with OFC-proven hazelnut allergy [25]. Among 123 Spanish children with suspected peanut allergy, the frequencies of sensitization to Ara h 1, Ara h 2, and Ara h 3 were 60.0%, 72.7% and 43.6% respectively, with significantly higher sIgE levels in the allergic group [26]. Among 40 Thai children with peanut sensitization, sIgE to Ara h 2 was a marker of anaphylactic reactions, whereas sIgE to Ara h 9 was unrelated to severe reactions [27]. Among 57 Japanese peanut-sensitized children, sIgE to Ara h 2 (cut-off 0.35 kU/l) could discriminate allergic from tolerant children with a sensitivity of 88% and a specificity of 84% [28].
- **Cow’s milk allergy -** Several studies have tried to determine the serum level cut-off of sIgE to cow’s milk useful to identify sensitized children who are at risk of developing allergic reactions to cow’s milk [14]. Among 123 Brazilian children with cow’s milk allergy (CMA), testing sIgE to whole cow’s milk extract (cut-off 3.06 kU/l) was more useful to diagnose CMA than testing sIgE to α-lactalbumin, β-lactoglobulin or casein [29]. Finally, SPT with milk protein may be more reliable than sIgE level in predicting outcomes in OFC with baked milk products [30].
- **Egg allergy -** SPT and sIgE to egg white have been shown to be a poor predictor of clinical phenotypes of egg allergy (EA). Among 154 Spanish egg-sensitized infants with CMA and/or atopic dermatitis without previous egg consumption, an egg white SPT wheal reaction of 8 mm and/or sIgE >8.36 KU/l predicted a positive outcome in OFC of around 94% [31]. Most children with EA seem to tolerate baked egg, but tolerance cannot be predicted with conventional allergy testing. In 186 English children with suspected EA who underwent an OFC, SPT to egg extract was slightly better in predicting a positive outcome than SPT to raw egg [32]. Egg white and yolk contain more than 20 different glycoproteins and measurement of sIgE to egg white subcomponents is considered as a new method for diagnosis [33]. Recently, it has been shown that sIgE to egg component Gal d 1 (i.e. ovomucoid) was more accurate in predicting raw EA compared with sIgE to egg white [34-36]. Moreover Gal d 1 negative children showed high frequency of tolerance to boiled egg [37]. In a prospective study of 143 children with EA, SPT to muffin < 2 mm had a high negative predictive value for baked egg OFC, whereas ovomucoid SPT 11 mm was very likely to predict a reaction to baked egg [38]. In 85 Spanish children sIgG4 to ovoalbumin resulted an independent predictor of tolerance development to uncooked egg [39].
- **Wheat allergy -** sIgE to wheat has poor diagnostic predictability for wheat allergy. A prospective study reported that wheat sIgE levels required to identify subjects with >95% probability of reacting to a wheat challenge were 80 kUA/L [14]. The association of sensitization to ω-5-gliadin with wheat-dependent exercise-induced anaphylaxis (EIA) is one of the best documented forms of wheat allergy [40]. A recent study of 108 Finnish children with suspected wheat allergy, who underwent open or double-blinded, placebo-controlled oral wheat challenges identified the “dimeric alpha-amylase inhibitors 0.19” as a relevant allergen in clinically reactive participants [41]. Cases of wheat-dependent EIA with positivity to nsLTP in absence of ω-5-gliadin sensitization have been reported [42]; thus, patients with a history consistent with cofactor-enhanced food allergic anaphylaxis should be tested for sIgE to nsLTP (e.g. Tri a 14) and to ω-5-gliadin [43].
- **Fish and seafood allergy -** The major codfish allergen component, Gad c 1 belongs to a group of Calcium-transporting muscle proteins known as parvalbumins. The variable degrees of clinical cross-reactivity in patients with fish allergy could be explained by the various degrees of amino acid homologies ranging from 60% to 80% shared by parvalbumins [44]. For shellfish allergy, tropomyosin is the major allergen responsible for cross-reactions among different species of the shellfish group. Molecular comparison of tropomyosin from different crustacean species revealed a very high homology of up to 98% [44]. It is well known that crustacean and mollusc allergens do not cross-react with fish allergens. On the other hand, patients with shellfish allergy are frequently reported to have allergic reactions to non-crustacean source, such as house dust mites and cockroaches. This cross-reactivity is probably due to the high amino acid homology of tropomyosins shared by these organisms [45]. Despite CRD revealed to be a useful tool to highlight in vitro-immunologic cross-reactivity in seafood allergy, its use in predicting clinical cross-reactivity is still to be improved.
- **Fruit and vegetable allergy** - Pru p 3 is an nsLTP and considered a major peach allergen component. In 57 Spanish children suffering from allergic reactions after eating or having contact with peach, sIgE to Pru p 3 was detected in 96% of participants but OFC with peach pulp showed that more than 90% tolerated peeled peach [46]. Allergic reactions to fruits and vegetables can either result from primary sensitization to food or to inhalant allergens. In the latter case triggering foods share similar components with inhalant allergens causing cross-reactions. A study performed on 15 subjects from Belgium with birch pollen allergy and suspected soy allergy, showed that secondary soy allergy may be responsible for chronic allergic symptoms (e.g. chronic severe generalized itching, recurrent urticaria, chronic diarrhea and generalized atopic dermatitis), besides typical immediate manifestations (from oral allergic syndrome up to anaphylaxis). In patients with birch pollen allergy SPT with soy flour and sIgE to soy component Gly m 4 were proposed as valuable tools for the diagnosis of secondary soy allergy [47]. Usually, cross reactivity is attributable to heat-labile allergens (i.e., PR-10 and profilins) and it is associated with mild oral reactions. On the contrary heat and proteolysis-resistant allergens (such as storage proteins and nsLTP) that primary sensitize through the oral route are associated with local and systemic reactions [48].

In a cohort of 30 children with IgE-mediated lentil allergy, initial lentil sIgE level < 4.9 kU/l correlated with significantly higher likelihood (68.4% vs. 18.2%) of outgrowing the lentil allergy than initial sIgE level ≥ 4.9 kU/l (p = 0.008). This findings suggests that sIgE levels may be important for predicting clinical reactivity and persistence of lentil allergy [49]. Reports of allergy to lupin are increasing with the diffusion of the lupin flour consumption in bakery due to primary sensitization or cross-reactions with other legumes. In a group of 12 Italian children allergic to peanut, b-conglutin has been identified as the major lupin allergen causing in vitro and in vivo cross-reactivity with peanut components [50].

Despite the CRD shows promise in reducing the need for OFC, there are circumstances in which OFC remains the only reliable method to ascertain the diagnosis of FA, such as cases of suspected food-dependent EIA with discordant medical history and sIgE results [51], or cases of suspected food protein-induced enterocolitis syndrome (FPIES), a non-IgE mediated FA in which *in vivo* and *in vitro* allergy tests show poor diagnostic accuracy. An OFC may not be necessary to diagnose FPIES only in very indicative cases (i.e. two or more acute episodes triggered by the same food in a six month period, with prompt resolution after avoidance of the causative food) [9].

### Food avoidance

Avoidance of the offending food and emergency treatment of adverse reactions are currently the mainstays for the management of IgE-mediated and non-IgE-mediated FA [8]. A dietary programme for FA should always include education on how to avoid specific allergens as well as comprehensive nutrition assessment on how to appropriately substitute foods in order to obtain adequate energy intake and nutrients for age [52]. Indeed, food avoidance poses the risk of malnutrition and nutrient deficiency in growing allergic children [53]. There is evidence that children with FA present a decreased weight-for-age and height-for-age compared to healthy subjects. Growth differences in children with FA are evident despite an appropriate caloric intake and correlate with the number of foods avoided and duration of the diet [54,55]. A recent US cross-sectional study confirmed a poor growth in children with FA (IgE- and non-IgE-mediated) supplemented with amino-acid formula, despite an adequate daily energy intake [56]. The mechanisms responsible for growth differences in children with FA are still unclear. Risk factors which may lead to growth deficit in such category of children seem to be the early onset of the disease, multiple food allergies, elimination of foods with high nutritional value (e.g. cow’s milk and egg) and subclinical intestinal inflammation with increased intestinal permeability [53]. In a recent prospective study conducted in 131 food allergic children on a restricted diet for cow’s milk and egg, about one-third of asymptomatic participants showed elevated intestinal permeability [57].

Another aspect that must be considered is that dietary elimination significantly affects the quality of life of allergic children and their families due to social restriction and risk of accidental reactions [58-60]. A Canadian study reported an annual incidence rate of 12.5% of accidental exposure to peanut in allergic children, with higher risk for adolescents and cases with recent diagnosis [61]. Questionnaires-based surveys on food allergic patients ‘quality of life addressed at different age groups, showed that food avoidance correlate with anxiety about an accidental adverse reaction [62,63]. Social occasions, trips and even only playing at friends’ houses are considered dangerous settings by parents of allergic children [64]. Shopping is considered a difficult activity because of the need to identify ingredients on food labels [52]. A restricted diet can even results in an economic burden for the family budget, especially in infants requiring amino-acid formula supplementation [65].

Finally, it must be considered that FA tends to resolve in most cases during the first years of life. Therefore the required period of strict elimination diet is not *a priori* established and periodical re-evaluations by the allergist are fundamental to assess the changing nutritional needs and eventually resolution of the disease.

### Specific oral tolerance induction: a promising alternative treatment

Over the last two decades, alternative treatment strategies have been investigated for FA, mainly targeting foods that commonly trigger IgE-mediated FA in children (i.e. cow’s milk, egg and peanut) [66]. As FA develops as a result of failure or loss of oral tolerance to food allergens, one of the most promising therapeutic approach pursued is oral immunotherapy or specific oral tolerance induction (SOTI) [67]. SOTI consists in oral assumption of increasing doses of the relevant allergen performed in controlled setting; this build-up phase is followed by a daily regular assumption of the tolerated dose which typically occurs at home. The aim is to induce an immune modulation in order to achieve a permanent oral tolerance [68].

There is a growing body of literature to show that SOTI is effective in increasing the threshold of reactivity for the most common triggering foods. This process, also referred as “desensitization”, can confer protection against accidental allergic reactions and can contribute to improve nutritional status and quality of life [69-79]. In a recent controlled trial conducted by Dello Iacono et al., 20 children with severe hen’s EA were equally randomized to receive SOTI with raw hen’s egg emulsion or egg-free diet for 6 months. At the end of the study period 90% of the SOTI group achieved partial tolerance, whereas sensitivity to raw hen's egg remained unchanged in 90% of controls [70]. Longo et al. reported that SOTI was effective in desensitizing a significant percentage of children with very severe CMA and high levels of sIgE [71]. Staden et al. investigated the efficacy of SOTI in comparison with the elimination diet in children with CMA or hen’s EA. At the follow-up challenge 36% in the SOTI group showed permanent tolerance, 12% were tolerant with regular intake and 16% were partial tolerant, whereas only 35% developed tolerance in the control group [72].

An open point of discussion is whether SOTI induces persistent tolerance or transient desensitization which may need regular allergen intake to be maintained [80]. Recent evidence suggests that SOTI to milk, egg and peanut can result in sustained unresponsiveness to those foods after discontinuing the maintenance dose, but more research into the long-term outcomes of SOTI is needed [76-79]. The immunological mechanisms underlying SOTI are currently unknown and their understanding could address the issue regarding long-term response to this treatment [68]. Fuentes-Aparicio et al. reported that acquisition of tolerance after SOTI in hen’s egg-allergic children is accompanied by a decrease in effector-memory CD4+ T-cell population and an increase in a hypo-proliferative subset of CD4+ T-cell population. This subset could represent a marker of tolerance induction [81]. Additionally, Vila et al. showed that the development of clinical tolerance to egg through SOTI is associated with a decrease in the mean sIgE level and in the antigen-specific basophil responsiveness [82].

Despite SOTI has demonstrated efficacy for clinical desensitization, one of the major concern is its safety. Adverse reactions occurring during SOTI are usually mild to moderate, predominantly oropharyngeal and easy to manage, but systemic severe events have been reported [83]. Therefore SOTI has still to be considered an experimental treatment belonging to the contest of clinical trials and further research is needed to establish its long-term efficacy, safety and cost-effectiveness [67]. An emerging strategy and possible alternative to SOTI for the treatment of IgE-mediated CMA and EA is the use of extensively heated forms of these allergens, such as in baked products. Recent studies have shown that the majority of children with CMA and EA tolerated baked milk or egg during an OFC and safely incorporated these products into their diet [34,84]. Tolerance of baked milk or egg is considered a marker of transient IgE-mediated FA [85].

### Anaphylaxis treatment

Prompt administration of intramuscular epinephrine is the first-line therapy for food-induced anaphylaxis [8]. Maintaining access to an adrenaline auto-injectors (AAI) as well as using an AAI are essential steps for an effective management of IgE-mediated FA [86]. First, it has to be decided who should be prescribed an AAI. However there are still no generally valid indication criteria for prescribing an AAI [87]. Johnson et al. showed the prescription practice of an AAI were very variable among pediatricians. The most influencing factors included peanut or tree nut allergy and parental anxiety [88]. So, as long as there are no improved anaphylaxis guidelines, the indication should be given individually considering the medical history and family’s needs [89]. The next step is defining when epinephrine should be used. The best outcome is provided when epinephrine is given as soon as early symptoms of anaphylaxis occur [87]. Hence, the focus should lie on early recognition of symptoms to promptly use the AAI. To this purpose Jacobs et al. conducted a survey to investigate the recognition and treatment of first initial food allergic reactions. They detected that only one-third of the initial reactions with symptoms likely to represent anaphylaxis were recognized and treated with epinephrine. Remarkably, only half of these patients treated with epinephrine were prescribed an AAI [90].

H1-antihistamines have long been used for mild, isolated, non-progressive cutaneous reactions to help relieving pruritus, hives, angioedema and conjunctivitis. However, H1- and H2-receptor antagonists cannot be used as substitute for epinephrine, but only as adjunctive medications during anaphylaxis [91]. In a recent EAACI systematic review on FA management, it has been reported a weak evidence (level of evidence III, grade C) to support the benefits of H1 antihistamines for children and adults with acute non-life-threatening symptoms of FA [8]. However no evidence for efficacy of antihistamines in the treatment of more severe symptoms was reported. Furthermore, the prophylactic administration of antihistamines can early mask symptoms of anaphylaxis and lead to delayed treatment of dangerous reactions with epinephrine. Inhaled beta-2 adrenergic agonists have been used for the relief of cough and wheezing in addition to epinephrine, but are not a substitute for epinephrine [91]. Oral or intravascular corticosteroids are often administered during an acute reaction to prevent protracted or biphasic episodes of anaphylaxis. Other interventions include supplemental oxygen and fluids which may be considered after the administration of epinephrine depending on the patient’s symptoms [92]. A recent Cochrane review was unable to identify relevant studies with evidence for the use of corticosteroids in anaphylaxis [93].

## Conclusion

The management of FA in children is improving through the acquisition of new knowledge in the field of diagnosis and treatment. CRD and SOTI are being intensively investigated. Their implementation in routine clinical practice can offer new perspectives in the management of patients with FA. In parallel, education of physicians and food allergic patients about anaphylaxis and its treatment is becoming recognized as an unmet need. The recent integration of all this new knowledge in international guidelines was a great achievement. The broad implementation of these guidelines all over Europe is now the new challenge for specialists, patients and other stakeholders of FA.

## Abbreviations

AAI: Adrenalin auto-injector
CMA: Cow’s milk allergy
CRD: Component resolved diagnosis
EA: Egg allergy
EIA: Exercise-induced anaphylaxis
FA: Food allergy
FPIES: Food protein-induced enterocolitis syndrome
nsLTP: Nonspecific lipid transfer protein
OFC: Oral food challenge
PR-10: Pathogenesis-related protein family
sIgE: Specific IgE
SPT: Skin Prick test
SOTI: Specific oral tolerance induction.
